# Interaction Between BGLF2 and BBLF1 Is Required for the Efficient Production of Infectious Epstein–Barr Virus Particles

**DOI:** 10.3389/fmicb.2019.03021

**Published:** 2020-01-24

**Authors:** Chien-Hui Hung, Ya-Fang Chiu, Wen-Hung Wang, Lee-Wen Chen, Pey-Jium Chang, Tsung-Yu Huang, Ying-Ju Lin, Wan-Ju Tsai, Chia-Ching Yang

**Affiliations:** ^1^Graduate Institute of Clinical Medical Sciences, Chang-Gung University, Taoyuan, Taiwan; ^2^Division of Infectious Diseases, Chang Gung Memorial Hospital Chiayi Branch, Chiayi, Taiwan; ^3^Department of Microbiology and Immunology, Chang-Gung University, Taoyuan, Taiwan; ^4^Research Center for Emerging Viral Infections, Chang-Gung University, Taoyuan, Taiwan; ^5^Department of Medical Laboratory, Chang Gung Memorial Hospital, Taoyuan, Taiwan; ^6^Division of Infectious Disease, Department of Internal Medicine, Kaohsiung Medical University Hospital, Kaohsiung Medical University, Kaohsiung, Taiwan; ^7^Department of Respiratory Care, Chang-Gung University of Science and Technology, Chiayi, Taiwan; ^8^Graduate Institute of Chinese Medical Science, China Medical University, Taichung, Taiwan

**Keywords:** BGLF2, BBLF1, maturation, final envelopment, acidic cluster

## Abstract

BGLF2 is a tegument protein of the Epstein–Barr virus (EBV). This study finds that BGLF2 is expressed in the late stage of the EBV lytic cycle. Microscopic investigations reveal that BGLF2 is present in both the nucleus and the cytoplasm and colocalized with BBLF1 and gp350 at juxtanuclear regions in the cytoplasm. This study also finds that the basic KKK_69_ motif of BGLF2 and acidic DYEE_31_ motif of BBLF1 are crucial for the interaction between BGLF2 and BBLF1, which is required for the recruitment of BGLF2 to the BBLF1 that is anchored on the *trans*-Golgi-network (TGN). In addition, BGLF2 in a density gradient is co-sedimented with un-enveloped capsids, revealing that BGLF2 associates with the EBV capsid before the final envelopment. The knockout of BGLF2 expression is demonstrated to reduce the numbers of infectious virions that are released into the culture medium, but they do not affect the expression of lytic proteins and viral DNA replication. The production of infectious viral particles by a BGLF2-knockout mutant can be rescued by exogenously expressed BGLF2 but only partially rescued by BGLF2-3KA, which is a mutant with reduced ability to interact with BBLF1 but does not affect its ability to activate the MAPK pathway and the expression of the EBV lytic proteins, suggesting that the interaction of BGLF2 with BBLF1 is important to the efficient production of infectious viral particles during the maturation. The results of this study improve our understanding of how BGLF2 promotes EBV viral production.

## Introduction

Epstein–Barr virus (EBV) causes infectious mononucleosis and is associated with Burkitt’s lymphoma, nasopharyngeal carcinoma, Hodgkin’s lymphoma, and gastric cancer (Epstein and Barr, 1964; Diehl et al., 1968; Klein et al., 1970; Takada, 2000). After infecting lymphoid and epithelial cells (Rickinson and Kieff, 2001), the virus is usually maintained under latent conditions, during which only a few latent genes are expressed (Pfeffer et al., 2004; Amon and Farrell, 2005). EBV is reactivated from latency to the lytic cycle by environmental stimulation to produce viral particles, although the stimuli that activate the EBV lytic cycle *in vivo* are unknown. Cells that are latently infected by EBV can be switched to the lytic cycle by treatment with 12-*O*-tetradecanoylphorbol-13-acetate (TPA), sodium butyrate, calcium ionophores, transforming growth factor, or anti-IgG (Zur Hausen et al., 1978; Faggioni et al., 1986; Daibata et al., 1990). During the immediate-early stage of the lytic cycle, EBV expresses two transcription factors, Rta and Zta (Countryman and Miller, 1985; Chevallier-Greco et al., 1986; Kolman et al., 1996; Feederle et al., 2000), which activate the transcription of lytic genes, ultimately enabling the virus to replicate its DNA from the lytic origin of replication (Hammerschmidt and Sugden, 1988; Carey et al., 1992; Ragoczy et al., 1998; Hung and Liu, 1999). Following DNA replication, the capsid proteins are made and EBV initiates an encapsidation process in the nucleus, during which the viral DNA is packaged (Sugimoto et al., 2019). The capsid then undergoes a maturation process, which involves primary envelopment, de-envelopment, and final envelopment (Skepper et al., 2001; Mettenleiter, 2004; Mettenleiter et al., 2009). The assembled capsids are recruited to the nuclear egress complex (NEC) at the nuclear inner membrane and bud into the perinuclear space to acquire an envelope during primary envelopment. This envelope is then removed by fusion with the outer nuclear membrane, releasing the capsid into the cytoplasm (Farina et al., 2005; Granato et al., 2008). According to studies on other herpesviruses, including herpes simplex virus type 1 (HSV-1), pseudorabies virus (Mettenleiter et al., 2009), human cytomegalovirus (HCMV) (Tandon and Mocarski, 2012), murine gammaherpesvirus-68 (MHV-68) (Peng et al., 2010), and Kaposi’s sarcoma-associated virus (KSHV) (Sathish et al., 2012), capsids in the cytoplasm are associated with tegument proteins and then recruited to the *trans*-Golgi-network (TGN)/endosomal-derived membrane (Wild et al., 2017; Nanbo et al., 2018). In the final envelopment process, tegumented capsids regain an envelope by budding into the TGN/endosomal-derived membrane, where the viral glycoproteins are located and enriched. The enveloped virion then exits the cell by exocytosis (Mettenleiter, 2004; Guo et al., 2010).

A mature virion of a herpesvirus comprises three morphologically distinct components, which are an icosahedral nucleocapsid, a lipid bilayer envelope with viral glycoproteins, and a proteinaceous layer, tegument, which occupies the space between the capsid and the envelope (Kalejta, 2008; Kelly et al., 2009; Sathish et al., 2012). The composition of the tegument in the virion of all three Herpesviridae subfamilies has been studied (Johannsen et al., 2004; Varnum et al., 2004; Zhu et al., 2005; Loret et al., 2008; Kramer et al., 2011). At least 17 virus-encoded tegument proteins are assembled into EBV virion (Johannsen et al., 2004). Tegument proteins play a key role in the maturation of EBV as well as the establishment of EBV during primary infection (Johannsen et al., 2004).

BGLF2 contains a Herpes_UL16 domain and is conserved among Herpesviridae (Chen et al., 1991, 2018). BGLF2 and its homologs, HSV-1 UL16, HCMV UL94, MHV-68, and KSHV ORF33, are present in the tegument layer of mature virions (Johannsen et al., 2004; Varnum et al., 2004; Loret et al., 2008; Guo et al., 2010). Deleting HCMV UL94 is known to abolish virion production (Phillips and Bresnahan, 2012). MHV-68 and KSHV ORF33 are crucial for virion morphogenesis and egress (Guo et al., 2009; Wu et al., 2015); deletion of UL11 or UL16 in HSV-1 reduces secondary envelopment during viral assembly and causes the accumulation of unenveloped nucleocapsids in the cytoplasm (Baines and Roizman, 1991, 1992; Starkey et al., 2014). Evidence suggests that the interaction between UL11 and UL16 provides a bridge between the nucleocapsid and the envelope during the final envelopment (Loomis et al., 2003; Meckes et al., 2010; Johnson and Baines, 2011). However, the involvement of BGLF2 in the final envelopment of EBV remains unclear. Recent results show that BGLF2 interacts with BKRF4 (Masud et al., 2017), and BGLF2 increases viral infectivity by activating AP-1 upon infection (Konishi et al., 2018). Additionally, BGLF2 induces G1/S arrest by increasing the p21 protein level (Paladino et al., 2014). The expression of BGLF2 activates mitogen-activated protein kinase (MAPK) signaling and promotes EBV lytic reactivation (Liu and Cohen, 2015). BGLF2 also inhibits NF-κB activity (Chen et al., 2019) and modulates JAK-STAT pathway (Sonia et al., 2019) to invade host antiviral innate immunity. These findings suggest that BGLF2 has an important role in the lytic cycle and primary infection.

This study finds that EBV BGLF2 is expressed in the late stage of the lytic cycle, associates with the un-enveloped capsid, and is recruited to the TGN membrane by interaction with BBLF1, a homolog of UL11. Knockout of the expression of BGLF2 reduces the production of infectious viral particles of EBV, which is rescued by exogenously expressed BGLF2 but partially rescued by BGLF2-3KA mutant, which exhibits a weaker interaction with BBLF1. These results suggest that BGLF2 is involved in the efficient production of infectious EBV through the interaction with BBLF1 during the maturation process.

## Materials and Methods

### Cell Lines and EBV Lytic Activation

HEK293 and HEK293T cells were maintained in DMEM that was supplemented with 10% fetal bovine serum (FBS). P3HR-1 was cultured in RPMI1640 medium that was supplemented with 10% FBS. P3HR1 cells were treated with 20 ng/ml TPA (Sigma) and 3 mM sodium butyrate (SB, Sigma) to activate the EBV lytic cycle. iD98HR1, a fusion cell between P3HR1 and an epithelial cell line D98, which expresses a Zta-estrogen receptor (Zta-ER) fusion protein (Nonoyama et al., 1978; Chiu et al., 2013), was cultured in DMEM that contained 10% FBS and treated with 200 nM 4-hydroxytamoxifen (OHT) to activate the EBV lytic cycle. A7, a melanoma cell line, was cultured in MEM that was supplemented with 10% FBS.

### Plasmids

Plasmids pBBLF1-Flag, pBBLF1-NDE-Flag, pBBLF1-SDE-Flag, pBBLF1-NDESDE-Flag, pGST-BBLF1, and pET32a-BBLF1 have been described elsewhere (Wang et al., 2011, 2015; Chiu et al., 2012). The BGLF2 gene was amplified with primers BGLF2-BXF 5′-TATAGGATCCCACGTCATGGCATCCGCCG and BGLF2-R 5′-ATCTCGAGCAATAAGAATGTAAGACCTGACG by polymerase chain reaction (PCR) using recombinant bacmid EBV p2089 DNA as a template. Plasmid pENTR3C-BGLF2 was constructed by inserting a PCR-amplified BGLF2 fragment into the *Bam*HI-*Xho*I sites of pENTR3C. Plasmid pGST-BGLF2, which expresses full-length BGLF2 that is fused to a glutathione *S*-transferase (GST) at its N terminus, was constructed by the transposition of a BGLF2 fragment from pENTR3C-BGLF2 into pDEST15 using the Gateway system (Invitrogen). The BGLF2 DNA fragment was also transposed into pcDNA-3.1/nV5-DEST and pDEST47 to yield pV5-BGLF2 and pBGLF2-GFP, respectively. The same strategy was used to construct plasmids that encode V5-tagged BGLF2 with its C-terminally deleted proteins. The primers that were used for amplification were BGLF2-BXF and BGLF2-328-R 5′-TATCTCGAGTCACAGTACTTGGCAATTGTAGAG, BGLF2-300-R 5′-TATCTCGAGTCACTCTTTCCCATTAGCAAGAAC, or BGLF2-155-R 5′-ATCTCGAGTTAGGGTGTGATATCTTGC AGTTC. A QuickChange site-directed mutagenesis kit (Agilent) was used to generate a 3KA mutation with pENTR3C-BGLF2. BGLF2 and BGLF2-3KA DNA were also transposed into pSLIK-zeo (Addgene) to generate pCMV-BGLF2 and pCMV-BGLF2-3KA, respectively.

### Immunofluorescence Staining

Cells were fixed with 4% paraformaldehyde and processed for indirect immunofluorescence staining, which was conducted using a primary antibody against Flag (Sigma), BBLF1 (Chiu et al., 2012), or BGLF2, following incubation with fluorescently labeled secondary antibodies (Invitrogen). Mouse anti-BGLF2 monoclonal antibody was generated using the synthesized peptide _145_KTVEELQDITPS_155_ (Abmart, China). To colocalize BGLF2 with EA-D or gp350, cells were first stained with anti-BGLF2 mAb, then incubated with fluorescently labeled secondary antibody, and finally incubated with fluorescently labeled anti-EA-D (Millipore) or anti-gp350 (Millipore) that had been labeled using a Zenon mouse IgG labeling kit (Invitrogen). Images were captured using a Leica SP5 fluorescence microscope.

### Immunoprecipitation and Immunoblot Analysis

Cells were lysed in PBS that contained 1% Triton X-100 and protease inhibitor cocktail (Sigma). Lysates were then cleared by centrifugation at 12,000 *g* at 4°C for 15 min. For immunoprecipitation, a specific antibody or GFP-Trap (ChromoTek) was added, and the mixture was incubated at 4°C for 1 h. Protein-A/G agarose beads (Upstate biotechnology) were added to capture the antigen-antibody complex. After washing with PBS that contained 1% Triton X-100, proteins that were bound to the beads were eluted by adding 2X electrophoresis sample buffer and analyzed by immunoblotting. For endogenous BGLF2 immunoblotting, cells were lysed in sample buffer that contained DTT. The primary antibodies were as follows: anti-Rta and anti-Zta (Argene), anti-EA-D, anti-gp110 and gp350 (Millipore), anti-Tubulin (Sigma), anti-GFP mAb (Roche), rabbit anti-V5 (Santa Cruz), anti-V5 mAb (Invitrogen), rabbit anti-V5 (GeneTex), anti p-ERK, ERK, p-p38, p38, p-JNK, JNK (Cell signaling), and rabbit anti-BGLF2 antibodies. Rabbit anti-BGLF2 antibody was produced using the synthesized peptide _21_LWVLSDASTPQMKV_34_-cys (AngeneBiotech, Taiwan).

### GST-Pulldown Assay

His-BBLF1, GST, and GST-BGLF2 proteins were expressed in *Escherichia coli* BL21 (DE3) and purified as described elsewhere (Chiu et al., 2012). After elution and quantification, His-BBLF1 was mixed with GST or GST-BGLF2 in PBS buffer that contained 1% NP40 and then separated into two tubes; GST-Sepharose beads were added to one tube, and Ni-beads were added to the other. After mixing at 4°C for 1.5 h, GST-Sepharose beads were washed extensively with PBS that contained 1% NP40; Ni-beads were washed with PBS that contained 1% NP40 and 0.2M imidazole. Proteins were eluted from the beads by adding 20 μl 2X electrophoresis sample buffer and were detected by immunoblotting with anti-6xHis and anti-GST antibodies (LTK Biolaboratories, Taipei, Taiwan).

### Enumeration of Virus Particles and EBV DNA Replication by qPCR

The amount of encapsidated viral DNA was determined by quantitative polymerase chain reaction (qPCR) using methods that were described elsewhere (Chiu et al., 2012; Hung et al., 2014). The samples were first treated with DNase I to remove genomic DNA and was followed by the treatment with SDS and proteinase K to remove the viral envelope and the capsid. EBV DNA was extracted using phenol–chloroform, precipitated with isopropanol, and recovered by centrifugation. The amount of EBV DNA was determined by qPCR using an iCycler iQ multicolor real-time PCR detection system (Bio-Rad) with primers and a probe that was specific to BKRF1 (EBV EBNA1 gene) (Ryan et al., 2004). The EBV lytic DNA replication was estimated by determining the number of copies of EBNA1 DNA in the total DNA preparation after normalization to the copy number of *PIK3CA*, which is a cellular gene with a single copy per cell. The number of the viral particles in nuclear or cytoplasmic subcellular fractions was determined by incubating cells in PBS that contained 0.5% NP-40 for 10 min on ice. Following low-speed centrifugation (1,000 *g* for 10 min), the supernatant was collected as the cytoplasmic fraction; the pellet was the nuclear fraction. Viral particles in the nuclear fraction were released by three rounds of freeze and thaw, followed by adding NP-40 at a final concentration of 2% (Shen et al., 2015). The nuclear lysate was subjected to centrifugation after incubation at 4°C overnight. The supernatant was collected, and the number of encapsidated EBNA1 copies therein was determined by qPCR, as described above.

### Isolation of Viral Particles

Viral particles were purified from 30 15-cm Petri dishes of iD98HR1 cells that had been treated with OHT for 3 days. The extracellular virions were collected from the culture supernatant. Cell debris was removed by centrifuging the supernatant at 6,000 *g* for 15 min. The intracellular viral particles were first released from cell pellets by three cycles of freeze and thaw. Viral particles in extracellular or intracellular fractions were concentrated by centrifugation at 130,000 *g* for 1 h on a 50% OptiPrep (Axis-Shield) cushion. The virions at the interface were collected, and the OptiPrep was adjusted to 25%. Subsequently, a gradient was generated by centrifugation at 350,000 *g* for 3 h with an NVT65 rotor (Beckman). Fractions of 1 ml were collected from the bottom of the tube. Proteins in each fraction were analyzed by immunoblotting with antibodies. Viral particles in the fractions were absorbed onto a formvar/carbon-coated grid (Ted Pella, Inc.), blotted dry, and negatively stained with 1% uranyl acetate for 15 min at room temperature. The morphology of the viral particles was examined, and images were obtained using a JEOL JEM-1200 transmission electron microscope.

### Construction of BGKF2KO Bacmid

The EBV mutant that lacked the BGLF2 gene was constructed by two-step Red-mediated mutagenesis in the GS1783 *E. coli* strain, as described elsewhere (Tischer et al., 2006). Primers 5′-ATATATTCTGGCACGTCATGGCATCCGCCGCGAACAGTA GCTGAAACGTCAGGTCTTACAAGGATGACGACGATAAGT AGGG and 5′-GCCACCTGCTTCAATAAGAATGTAAGACCT GACGTTTCAGCTACTGTTCGCGGCGGATGCCAACCAATT AACCAATTCTGATTAG were used to amplify a PCR product from pEP-Kan-S to generate a linear fragment that contained a kanamycin-resistant expression cassette, an I-SceI restriction enzyme site, and flanking sequences that were derived from EBV genomic DNA sequences, which included a 40-bp copy of the duplicated sequence. The fragment was then introduced by electroporation into GS1783 cells that harbored EBV Bacmid p2089, and integrated into EBV Bacmid p2089 by homologous recombination, to delete BGLF2. A second Red-mediated recombination between duplicated sequences removed the Kan/I-SceI cassette, leaving the sequences that overlapped the BGLF1 and BGRF1/BDRF1 coding sequence. Restriction fragment analysis was performed to verify the integrity of each recombinant BAC clone with *Bam*HI. Additionally, a PCR fragment that spanned the deleted region of BGLF2 was amplified and sequenced to confirm sequences of the deletion clone. The bacmid DNA from the established stable clone in HEK293 was also confirmed by extraction from cells, electroporated into DH10B, and analyzed by restriction fragment analysis.

### Reverse Transcription-Quantitative Polymerase Chain Reaction

HEK293 (2089) and HEK293 (del-BGLF2) were transfected with a control vector or pCMV-Zta. Total RNA was extracted after 24 h post-transfection using Easy Prep RNAprep purification kit (Tools Biotechnology). RNA was reverse-transcribed into cDNA using the SuperScript III first-strand synthesis supermix (Invitrogen). One hundred nanograms of cDNA was used in PCR that was performed using a Bio-Rad CFX apparatus. Primer sets used for PCR amplification are as follows: BGLF1: 5′-GCTGTCATCCTGTGCAGTCT and 5′-CT CGAGGGTGCCTGTTTCTT; BGLF2: 5′-CTCAGATGCAGTG GGTGAGG and 5′-GGCGGTTGAGGTGGTAAAGA; BGLF3: 5′-TCCAAGGGAGTGGGTCTTC and 5′-GCAGTTGGTGGA CAAGGTT; BGRF1/BDRF1: 5′-CATTACCCATGTTCGGTG CG and 5′-TAACGTGTCGCGTAGCTCTG; GAPDH: 5′-TCA TGGGTGTGAACCATGAGAAG and 5′-CAGTGATGGCATGG ACTGTGGTC.

### Infectivity of EBV

Culture supernatant was collected from HEK293 cells that carried EBV bacmid on day 3 post-transfection. Raji (1 × 10^6^) cells were then infected with 500 μl collected culture supernatant. Cells were then treated with TPA (20 ng/ml) and butyrate (3 mM) on day 2 post-infection to enhance the expression of the green fluorescent protein (GFP). The percentage of cells that expressed GFP was determined by flow cytometry on day 3 post-infection.

### Electron Microscope

HEK293 (p2089) and HEK293 (del-BGLF2) cells were transfected with pCMV-Zta to induce the lytic cycle. On day 3 post-transfection, viral particles in the culture media were harvested and were concentrated by ultracentrifugation. The pellet of viral particles was fixed in 2% paraformaldehyde and 2.5% glutaraldehyde for 30 min at 4°C. The samples were washed and post-fixed in 1% osmium tetroxide for 15 min and then stained with 1% uranyl acetate for 1 h at room temperature. Samples were dehydrated using increasing concentrations of ethanol from 50 to 100% and then embedded in Spurr resin. Embedded samples were sliced into thin sections and stained with uranyl acetate and lead citrate. Images of the samples were obtained using JEOL JEM-1200 transmission electron microscope.

### Statistical Analysis

Data are presented as means ± standard deviations (SD). Student’s *t*-test was conducted on these means, and a *p*-value of less than 0.05 was considered to indicate significance.

## Results

### Expression and Localization of BGLF2

**FIGURE 1:**
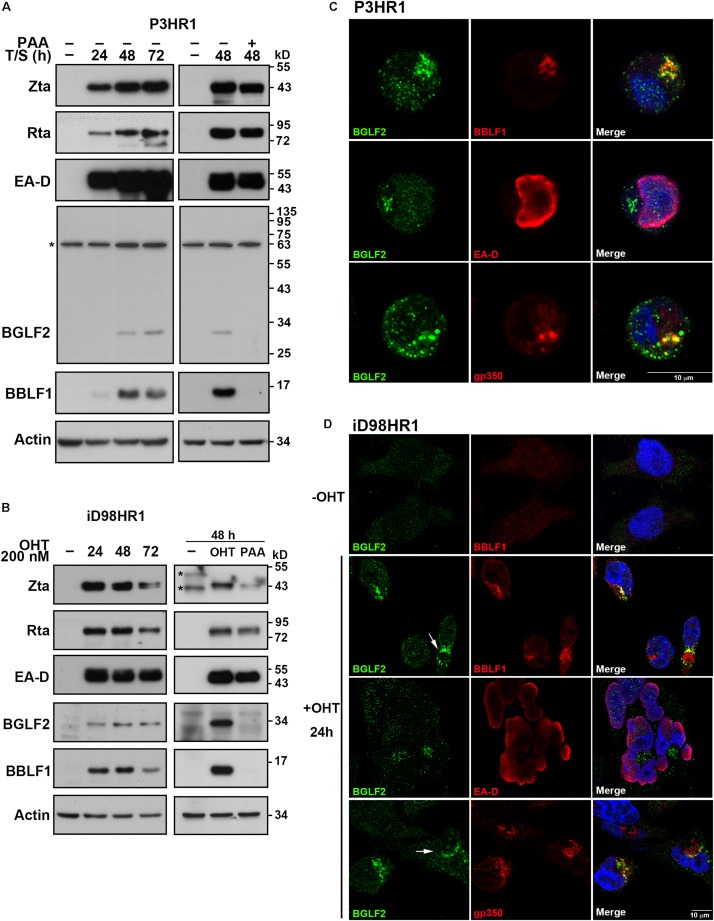
Expression and localization of BGLF2. **(A)** P3HR1 cells were treated or untreated with phosphonoacetic acid (PAA) at the time of lytic induction with 12-*O*-tetradecanoylphorbol-13-acetate (TPA) and sodium butyrate (SB) (T/S). The expression of Zta, Rta, EA-D, BGLF2, BBLF1, and actin by the cells was examined by immunoblotting at 0, 24, 48, and 72 h after lytic induction. *Non-specific band. **(B)** Epstein–Barr virus (EBV) in iD98HR1 cell was lytically activated by treatment with 4-hydroxytamoxifen (OHT) in the absence or presence of PAA. Cell lysates were prepared after lytic induction and analyzed by immunoblotting using antibodies, as indicated. *Non-specific band. **(C,D)** P3HR1 and iD98HR1 were treated with TPA and SB, and OHT for 48 and 24 h, respectively, to activate the EBV lytic cycle. Cells were fixed and stained with indirect immunofluorescence using anti-BGLF2, anti-EA-D, anti-BBLF1, or anti-gp350 antibodies and were then incubated with fluorescently labeled secondary antibodies. DAPI staining (blue) revealed the nucleus. Arrow: BBLF1 and gp350 staining that was surrounded by BGLF2. Images were captured and analyzed using a Leica SP5 confocal laser-scanning microscope.

The expression of EBV lytic proteins by P3HR1 cells was examined by immunoblot analysis after lytic induction with SB and TPA. EBV immediate-early and early proteins, including Rta, Zta, and EA-D, were detected at 24 h after lytic induction. However, the maximal expression of BBLF1 and BGLF2 was detected at 48 h after lytic induction (Figure 1A). The expression of EBV lytic protein by iD98HR1 cells was also examined after lytic induction with OHT. Unlike in P3HR1 cells, the expression of BBLF1 and BGLF2 in iD98HR1 was detected as early as 24 h after lytic induction (Figure 1B). The overexpressed Zta-ER may have caused the expression of BGLF2 at 24 h rather than at 48 h after lytic induction, as in P3HR1 cells. The expression of BBLF1 and BGLF2 was unobserved when the cells were treated with phosphonoacetic acid (PAA), which is an EBV DNA polymerase inhibitor (Figures 1A,B), at the time of lytic induction, indicating that the expression of BBLF1 and BGLF2 depends on lytic DNA replication and that BBLF1 and BGLF2 are EBV late proteins. Meanwhile, the localization of BGLF2 in P3HR1 and iD98HR1 cells was examined by immunofluorescence staining (Figures 1C,D). BGLF2 was detected in iD98HR1 only after OHT treatment, suggesting that BGLF2 was specifically recognized by the antibody (Figure 1D; first row). Meanwhile, BGLF2 was found to be localized in the nucleus and cytoplasm at 24 h after lytic activation, and the nuclear staining was more prominent in P3HR1 than in iD98HR1 cells (Figures 1C,D). BGLF2 was localized in the juxtanuclear area in the cytoplasm (Figures 1C,D) and found to be partially colocalized with BBLF1 and gp350 in P3HR1 (Figure 1C; first and third rows) and iD98HR1 (Figure 1D; second and fourth rows) cells. In iD98HR1 cells, juxtanuclear staining of BGLF2 was not always observed in BBLF1- and gp350-positive cells. In some cells, BGLF2 surrounded BBLF1 and gp350 staining (Figure 1D; arrows). These results suggest that BGLF2 is a true late protein; its distribution is variable, and BGLF2 may have both nuclear and cytoplasmic functions.

### Recruitment of BGLF2 to *TGN* by Interaction With BBLF1

**FIGURE 2:**
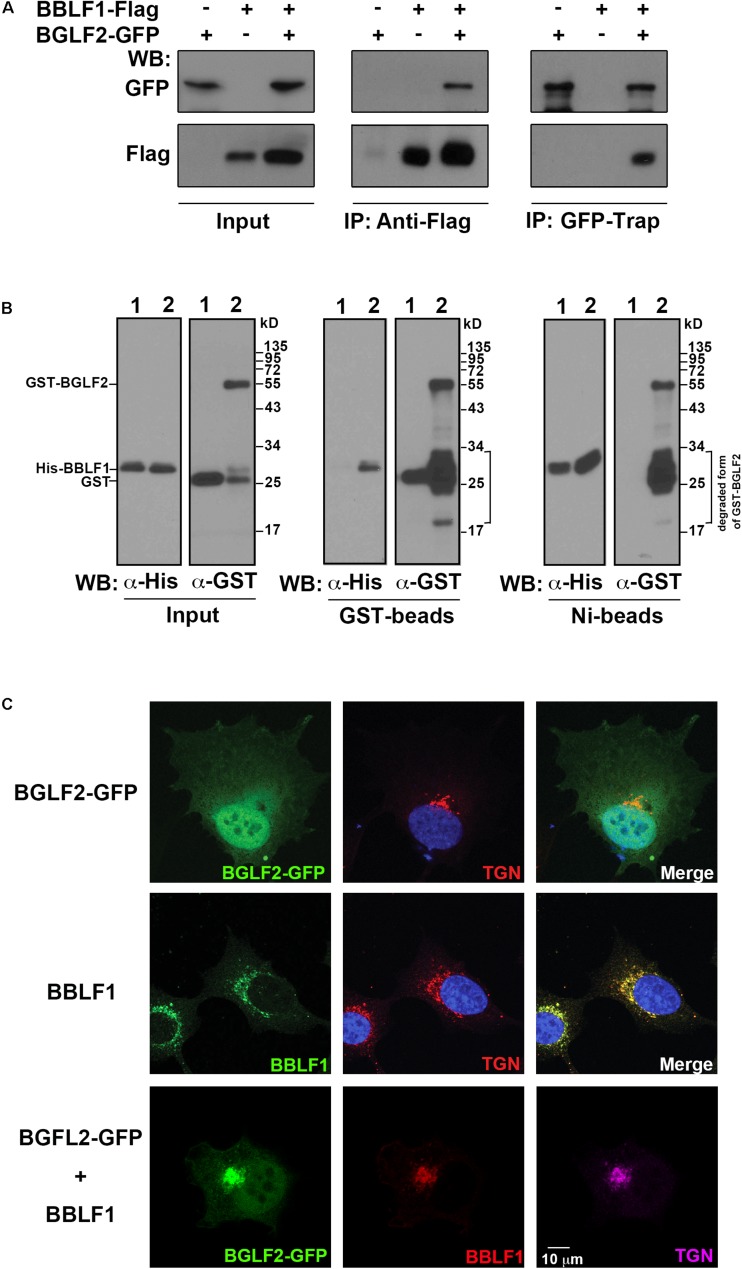
Interaction between BBLF1 and BGLF2. **(A)** 293T cells were transfected with pBGLF2-GFP or pBBLF1-Flag or cotransfected with pBGLF2-GFP and pBBLF1-Flag. At 48 h after transfection, proteins in the lysates were immunoprecipitated (IP) with anti-Flag antibody or precipitated using GFP-Trap and then analyzed by immunoblotting (WB) with anti-GFP or anti-Flag antibodies, as indicated. Input lanes were loaded with 2% of the lysates. **(B)** His-BBLF1, GST, and GST-BGLF2 were expressed in *Escherichia coli* and purified using Ni-NTA-agarose beads or glutathione-Sepharose beads. His-BBLF1 was mixed with GST (lane 1) or GST-BGLF2 (lane 2). Proteins in the mixture were detected by immunoblot analysis using anti-His and anti-GST antibodies (Input). Glutathione-Sepharose (GST-beads, middle panel) and Ni-NTA-agarose beads (Ni-beads, right panel) were added to the mixture. Proteins that were bound to the beads were analyzed by immunoblot analysis using anti-His and anti-GST antibodies, as indicated. Input lanes were loaded with 4% of purified proteins. **(C)** A7 cells were transfected with a plasmid that expresses BGLF2-GFP (upper row) or BBLF1-Flag (middle row) or cotransfected with plasmids that express BGLF2-GFP and BBLF1-Flag (lower row). Cells were fixed at 48 h post-transfection. Indirect immunofluorescence staining was performed using anti-BBLF1 and anti-TGN46 antibodies and was followed by incubation with fluorescently labeled secondary antibodies. DAPI staining (blue) revealed the nuclei. Images were captured and analyzed using a Leica SP5 confocal laser-scanning microscope.

BGLF2 is present in the tegument layer of the EBV virion (Johannsen et al., 2004). Since HSV-1 UL16, a homolog of BGLF2, interacts with UL11 (Loomis et al., 2003), which is a homolog of EBV BBLF1, whether BGLF2 interacted with BBLF1 was examined. Lysates were prepared from HEK293T cells that had been transfected or cotransfected with pBGLF2-GFP and pBBLF1-Flag. Immunoblot analysis revealed that anti-Flag antibody immunoprecipitated BBLF1-Flag and coimmunoprecipitated BGLF2-GFP; GFP-Trap precipitated BGLF2-GFP and coprecipitated BBLF1-Flag (Figure 2A), demonstrating that BGLF2 interacted with BBLF1 *in vivo*. Bacterially expressed GST or GST-BGLF2 was incubated with purified His-BBLF1 (Figure 2B; lanes 1 and 2). The results showed that His-BBLF1 was pulled down by GST-BGLF2-glutathione-Sepharose beads, but not by GST-glutathione-Sepharose beads (Figure 2B; GST-beads). Similarly, GST-BGLF2, but not GST, was captured by His-BBLF1 that was bound to Ni-NTA beads (Figure 2B; Ni-beads). These results reveal that BGLF2 interacted directly with BBLF1. After pull-down assay, the amounts of proteins around 24–34 kDa were increased compared to that in input, suggesting GST-BGLF2, in which GST is fused to the N-terminus of BGLF2, was unstable (Figure 2B; lane 2 in GST-Beads and Ni-beads).

Since the coimmunoprecipitation assay revealed that BBLF1 interacts with BGLF2 (Figures 2A,B), whether BBLF1 affected the localization of BGLF2 was examined. Unlike BBLF1, which is known to localize at *TGN* (Figure 2C; middle row) (Chiu et al., 2012), BGLF2-GFP was found to be present in both the nucleus and the cytoplasm but did not colocalize with a TGN marker, TGN46, in A7 cells (Figure 2C; upper row). However, when BBLF1 was coexpressed, BGLF2-GFP was found to colocalize with BBLF1 and TGN46 (Figure 2C; lower panel), suggesting that BBLF1 recruits BGLF2 to the *TGN* membrane, although nuclear and cytosolic distributions of BGLF2-GFP remained evident (Figure 2C).

### Involvement of Acidic Motifs in BBLF1 in the Recruitment of BGLF2 to TGN

**FIGURE 3:**
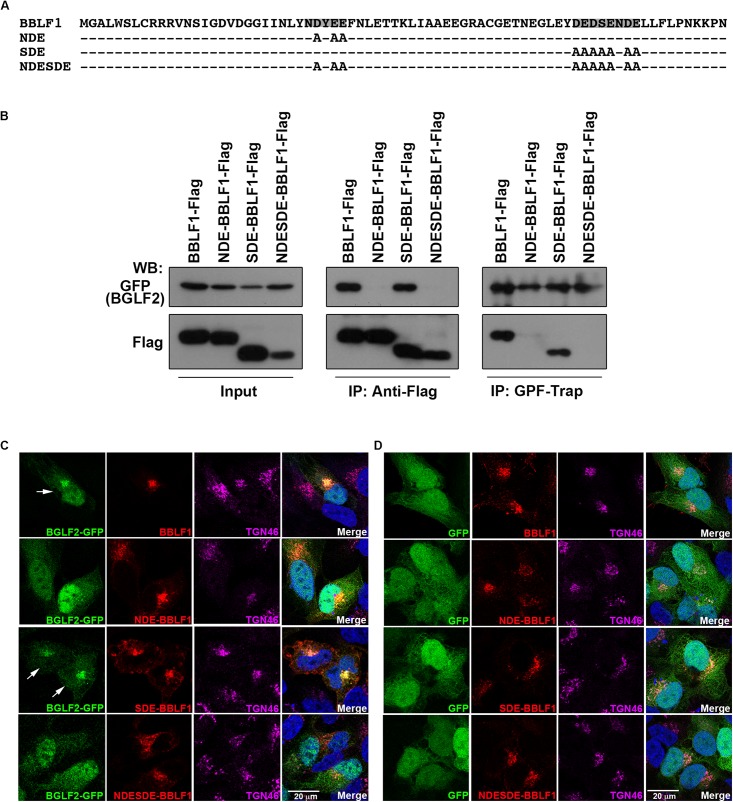
Interaction between the acidic cluster motifs in BBLF1 and BGLF2. **(A)** Sequences of BBLF1 and NDE-BBLF1, SDE-BBLF1, and NEDSDE-BBLF1. Acidic clusters of BBLF1 are highlighted. **(B)** 293T cells were cotransfected with pBGLF2-GFP and pBBLF1-Flag or a plasmid that expressed BBLF1 with an NDE, SDE, or NDESDE mutation. Proteins in the lysates were immunoprecipitated (IP) at 48 h post-transfection using anti-Flag antibody or precipitated using GFP-Trap and analyzed by immunoblotting with anti-GFP or anti-Flag antibodies, as indicated. A7 cells were cotransfected with plasmids that express BGLF2-GFP **(C)** or GFP **(D)** and BBLF1 or its mutant derivatives. Cells were fixed at 48 h post-transfection. Indirect immunofluorescence staining was performed using anti-BBLF1 and anti-TGN46 antibodies and was followed by incubation with fluorescently labeled secondary antibodies. DAPI staining (blue) revealed the nuclei. Images were captured and analyzed using a Leica SP5 confocal laser-scanning microscope. Arrow: BGLF2-GFP was recruited to TGN.

BBLF1 contains two acidic cluster motifs, NDYEE_31_ (NDE) and DEDSENDE_65_ (SDE) (Figure 3A), which participate in the retrograde transport of BBLF1 to *TGN* (Chiu et al., 2012). To determine whether these motifs in BBLF1 are involved in the interaction with BGLF2, HEK293T cells were cotransfected with plasmids that express BGLF2-GFP and BBLF1-Flag or its mutant derivatives with mutated NDE, SDE, and both NDE and SDE motifs, NDE-BBLF1-Flag, SDE-BBLF1-Flag, and NDESDE-BBLF1-Flag, respectively (Figure 3A). Immunoblotting revealed that BGLF2-GFP was coimmunoprecipitated with BBLF1-Flag and SDE-BBLF1-Flag, but not with NDE-BBLF1-Flag or NDESDE-BBLF1-Flag, by anti-Flag antibody (Figure 3B; middle panel). On the other hand, BBLF1-Flag and SDE-BBLF1-Flag, but not NDE- or NDESDE-BBLF1-Flag, were coprecipitated with BGLF2-GFP by GFP-Trap (Figure 3B; right panel), suggesting the NDE but not the SDE motif of BBLF1 was involved in the interaction with BGLF2. Meanwhile, this study cotransfected A7 cells with plasmids that express BGLF2-GFP and BBLF1. Confocal laser scanning microscopy revealed that these two proteins colocalized with TGN46 (Figure 3C; first row). Whether SDE-BBFL1, NDE-BBLF1, and NDESDE-BBLF1 mutants could recruit BGLF2 to TGN was also examined. The SDE mutation in BBLF1 did not affect the colocalization of BGLF-GFP with TGN46 (Figure 3C; third row). However, the NDE and NDESDE mutations of BBLF1 reduced the recruitment of BGLF2-GFP to TGN (Figure 3C; second and fourth rows). About 70% of transfected cells showed that BGLF2-GFP is recruited to TGN by BBLF1 or BBLF1-SDE. A parallel experiment showed that BBLF1 and its derivatives were unable to recruit GFP to TGN (Figure 3D). BGLF2-GFP or GFP also did not influence the colocalization of BBLF1 or its derivatives with TGN46 (Figures 3C,D). These results indicated that the interaction and recruitment of BGLF2 to TGN depend on interaction with the NDE acidic motif in BBLF1.

### Delineating the Region in BGLF2 That Interacts With BBLF1

**FIGURE 4:**
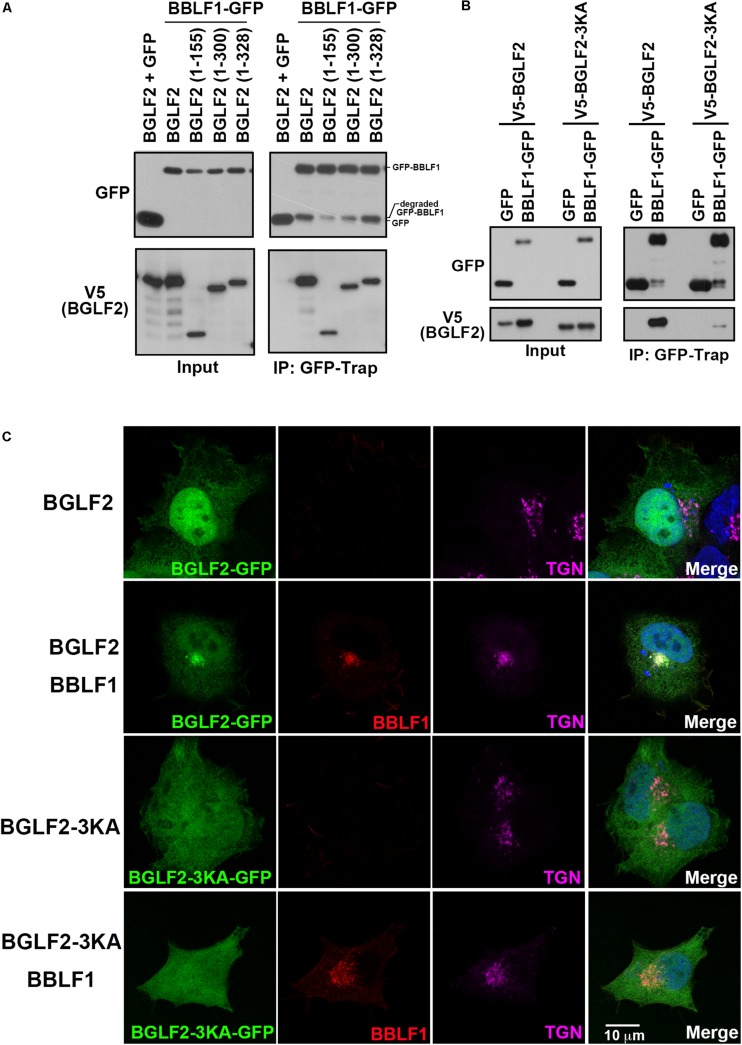
The BGLF2 region that interacts with BBLF1. **(A)** 293T cells were cotransfected with plasmids that express BBLF1-GFP and V5-BGLF2 or its deletion mutants. Proteins in the lysates were precipitated using GFP-Trap and analyzed by immunoblotting with anti-GFP and anti-V5 antibodies at 48 h post-transfection. Input lanes were loaded with 0.8% of the lysate. **(B)** 293T cells were cotransfected with pEGFP or pBBLF1-GFP and pV5-BGLF2 or pV5-BGLF2-3KA. Proteins in the lysates were immunoprecipitated (IP) using GFP-Trap and analyzed at 48 h post-transfection by immunoblotting using anti-GFP or anti-V5 (BGLF2) antibodies, as indicated. **(C)** A7 cells were transfected with a plasmid that expressed BGLF2-GFP (first row) or BGLF2-3KA-GFP (third row) or cotransfected with one of these plasmids with BBLF1-Flag (second and fourth rows) for 48 h. Cells were fixed, and indirect immunofluorescence staining was performed using anti-Flag and anti-TGN46 antibodies, it was followed by incubation with fluorescently labeled secondary antibodies. DAPI staining (blue) revealed the nucleus. Images were captured and analyzed using a Leica SP5 confocal laser-scanning microscope.

BGLF2 was deleted from its C-terminus to determine the region in BGLF2 that interacts with BBLF1. Our results showed that deleting the C-terminal region in V5-BGLF2, from aa 328, 300, 155 to 336, reduced the protein stability as these mutant proteins were less abundant than WT BGLF2 in the lysate (Figure 4A; input). Despite the instability, these proteins were coprecipitated with GFP-BBLF1 by GFP-Trap (Figure 4A; GFP-Trap), showing that the region between amino acids 1 and 155 in BGLF2 interacted with BBLF1. An attempt was made to delete the N-terminal 155 amino acids in BGLF2 to verify the interaction, but BGLF2 without its N-terminal 155 amino acids was found to be extremely unstable, preventing us from studying N-terminally deleted BGLF2. According to our sequence analysis, BGLF2 and its homologs in herpesviruses, although it does not share extensive sequence homology in the N-terminal region, all contain a basic amino acid motif, which in BGLF2 is KKK_69_. This motif was substituted with alanine to generate the 3KA mutant, BGLF2-3KA. This mutation did not affect protein abundance, although the mutant protein migrated in a gel faster than the wild-type BGLF2 (Figure 4B; input). Coimmunoprecipitation assay revealed that the binding between BBLF1 and BGLF2-3KA was substantially weaker than that between BBLF1 and WT BGLF2 (Figure 4B). A7 cells were transfected with plasmid pBGLF2-GFP or pBGLF2-3KA-GFP, and immunofluorescence staining showed that both BGLF2 and BGLF2-3KA localized in the nucleus and cytoplasm (Figure 4C; first and third rows). The nuclear staining of BGLF2-3KA was considerably weaker than that of BGLF2, suggesting the KKK_69_ motif may function as a nuclear localization signal. Furthermore, when A7 cells were cotransfected with plasmids pBBLF1-Flag and pBGLF2-GFP or pBGLF2-3KA-GFP, BGLF2 was redistributed to the TGN by BBLF1 (Figure 4C; second row), whereas TGN localization of BGLF2-3KA was undetected in the presence of BBLF1 (Figure 4C; fourth row). These results were consistent with those in the coimmunoprecipitation experiment, indicating that the basic amino acid motif in the N-terminal of BGLF2 is important to its interaction with BBLF1.

### Association of BGLF2 With the EBV Capsid

**FIGURE 5:**
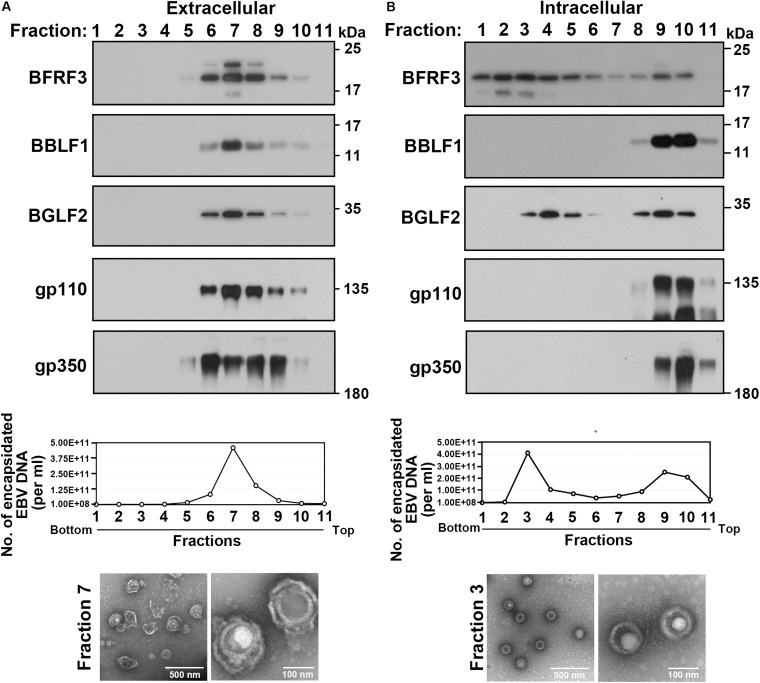
Association of BGLF2 with Epstein–Barr virus (EBV) capsids in the cytoplasm. **(A,B)** iD98HR1 was treated with 4-hydroxytamoxifen (OHT) for 3 days. Culture media **(A)** and cell pellets were harvested, and viral particles were concentrated by ultracentrifugation using a 50% OptiPrep cushion. The virus, which was in the interface, was collected. The samples that contained the virus were then ultracentrifuged through a gradient, which was self-generated during centrifugation with 25% OptiPrep. After ultracentrifugation and fractionation, proteins in each fraction were analyzed by immunoblotting with antibodies, as indicated. Encapsidated EBV DNA in each fraction was purified and quantified by real-time PCR (graph). Electron micrographs of enveloped virions (extracellular fraction 7) and non-enveloped capsids (intracellular fraction 3) are shown at the bottom of the panel.

To investigate the role of BGLF2 during EBV lytic cycle, this study purified extracellular and intracellular viral particles from iD98HR1 cells that had been treated with OHT. The viral particles in the culture medium were concentrated and then ultracentrifuged at 350,000 *g* for 3 h through a gradient that was formed with 25% OptiPrep. After fractionation of the gradient, proteins in each fraction were analyzed by immunoblotting. We found that BRFR3, BBLF1, BGLF2, gp110, and gp350 were found in fractions 6–9 (Figure 5A). Encapsidated EBV DNA was detected by qPCR in these fractions (Figure 5A; graph). Electron microscopy revealed the presence of EBV capsid that was wrapped by an envelope (Figure 5A; bottom). This result is consistent with the fact that both BGLF2 and BBLF1 are present in the tegument layer of mature virions (Johannsen et al., 2004). Intracellular EBV particles were also purified by gradient centrifugation. qPCR revealed the presence of EBV DNA in fractions 2–5, 9, and 10 (Figure 5B), suggesting that two types of viral particles were isolated from the cells. BBLF1, gp110, and gp350 were absent from fractions 2–5 but present in fractions 9 and 10 (Figure 5B), suggesting that fractions 2–5 contained unenveloped capsids, whereas fractions 9 and 10 contained membrane-associated capsids or enveloped virions. The viral particles in fraction 3 were further examined by TEM, and the micrographs revealed capsids with icosahedral morphology (Figure 5B; bottom). BGLF2 was detected in fractions 2–5, which contained unenveloped capsids, suggesting that BGLF2 associates with capsid before the final envelopment.

### BGLF2 and EBV Lytic Development

**FIGURE 6:**
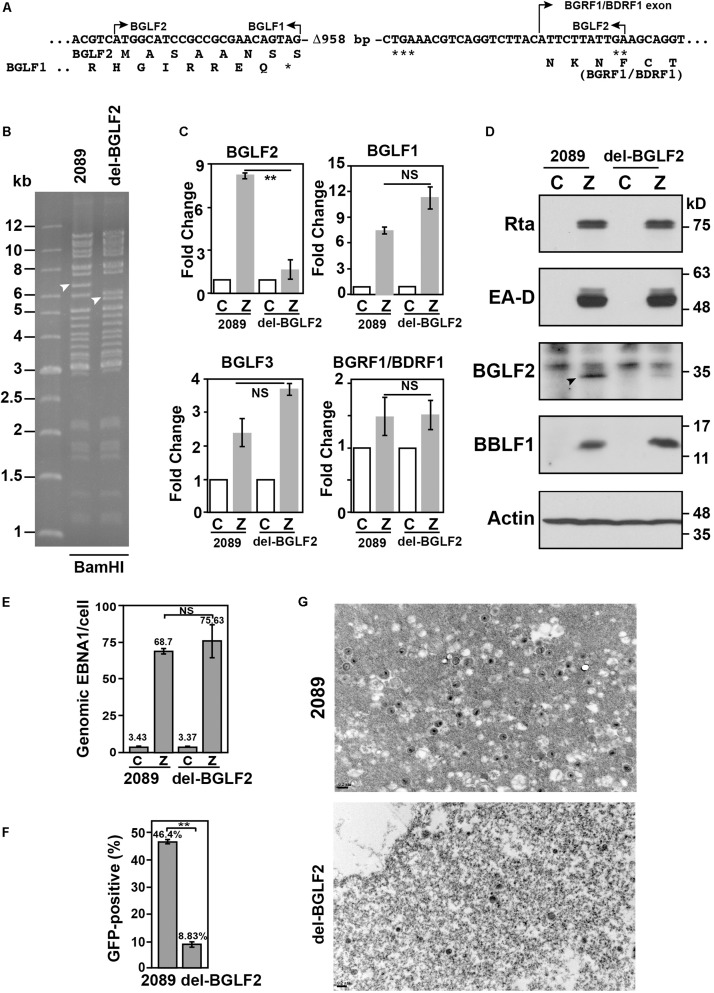
Influence of BGLF2 and Epstein–Barr virus (EBV) maturation. **(A)** Sequence of del-BGLF2. del-BGLF2 was generated by deleting an internal 958-bp sequence (Δ-958) in BGLF2. The deletion caused a premature termination at a termination codon that is indicated by ***. ** Indicates the termination codon of BGLF2. * indicates the termination codon of BGLF1. The amino acids encoded by the genes are shown below the DNA sequence. Arrows indicate the start and the end of an open reading frame, or BGRF1 exon. **(B)** p2089 and del-BGLF2 bacmid DNA were digested with *Bam*HI, and restriction fragments were analyzed with a 0.6% agarose gel. Arrowheads indicate the DNA fragment containing the BGLF2 sequence. **(C)** The expressions of BGLF1, BGLF2, BGLF3, and BGRF1/BDRF1 in HEK293 (2089) and HEK293 (del-BGLF2) were determined by RT-qPCR 24 h post-transfection. The relative expression of each gene in cells transfected with pCMV-Zta (Z) was normalized to the amount of GAPDH and expressed as the fold change relative to that cell transfected with empty vector (C). **(D)** At 72 h post-transfection, Rta, EA-D, BGLF2, BBLF1, and α-actin in the cell lysate were analyzed by immunoblotting. **(E)** The amount of EBV DNA in the cell lysates was analyzed by qPCR and was normalized to the number of copies of a cellular gene, PIK3CA. **(F)** Raji cells were infected with virus particles that were released by HEK293 (p2089) and HEK293 (del-BGLF2) cells after lytic induction by transfecting cells with pCMV-Zta. The percentage of Raji cells that were GFP-positive was determined by FACS analysis. The experiments were conducted three times, and the results were analyzed statistically using the *t*-test. **p < 0.01; NS, not statistically significant. **(G)** Electron micrographs of pelleted viral particles that were released by HEK239 (2089) and HEK293 (del-BGLF2) transfected with pCMV-Zta.

To determine the role of BGLF2 in EBV lytic development, a 958-bp fragment was deleted from BGLF2 in EBV bacmid p2098, generating a mutant, del-BGLF2 (Figure 6A). Although the BGLF2 sequence overlaps BGLF1 and BGRF1/BDRF1, the deleted region in BGLF2 did not extend into these two genes (Figure 6A). Restriction analysis showed that digesting p2098 DNA with *Bam*HI produced a 6.5-kb *Bam*HI-G fragment; however, the *Bam*HI digestion of del-BGLF2 DNA caused the disappearance of the 6.5-kb fragment but yielded a 5.6-kb fragment (Figure 6B; arrowhead), showing that a deletion in BGLF2 was indeed generated. The expressions of genes around BGLF2 locus, BGLF1, BGLF2, BGLF3, and BGRF1/BDRF1, were examined by reverse transcription quantitative polymerase chain reaction (RT-qPCR). The expression of BGLF2 was diminished in HEK293 (del-BGLF2), but the expressions of BGLF1, BGLF3, and BGRF1/BDRF1 were unaffected or even higher after 958-bp deletion in BGLF2 gene (Figure 6C). The results suggest that the deletion in del-BGLF2 did not affect the expression of adjacent genes. Immunoblotting revealed that HEK293 (p2098) but not by HEK293 (del-BGLF2) cells expressed BGLF2, but the expression of Rta, EA-D, and BBLF1 was unaffected by the deletion after EBV lytic activation (Figure 6D). Analysis of EBV DNA inside the cell by qPCR revealed that EBV DNA replicated and its amounts increased substantially upon lytic induction (Figure 6E). BGLF2 deletion did not influence this increase (Figure 6E), showing that deletion in BGLF2 did not affect the ability of EBV to replicate its DNA. The viral particles in the culture medium were also used to infect Raji cells. Green fluorescence exhibited by the Raji population after the infection was then determined by FACS on day 3 after infection. The results showed that p2089 EBV infected 46% of the Raji population, whereas the del-BGLF2 EBV infected only 9% of the population (Figure 6F), showing that the BGLF2 deletion significantly reduced the infectivity of EBV. Furthermore, EBV virions in the culture medium were concentrated by ultracentrifugation after lytic induction, and the number of viral particles was examined by electron microscopy (Figure 6G). These results suggest that BGLF2 deletion significantly reduced the production of mature virions that were released from the cells (Figure 6G), which may contribute to the reduced viral infectivity of del-BGLF2.

### Interaction Between BGLF2 and BBLF1 Is Important to the Efficient Production of Infectious Viral Particles

**FIGURE 7:**
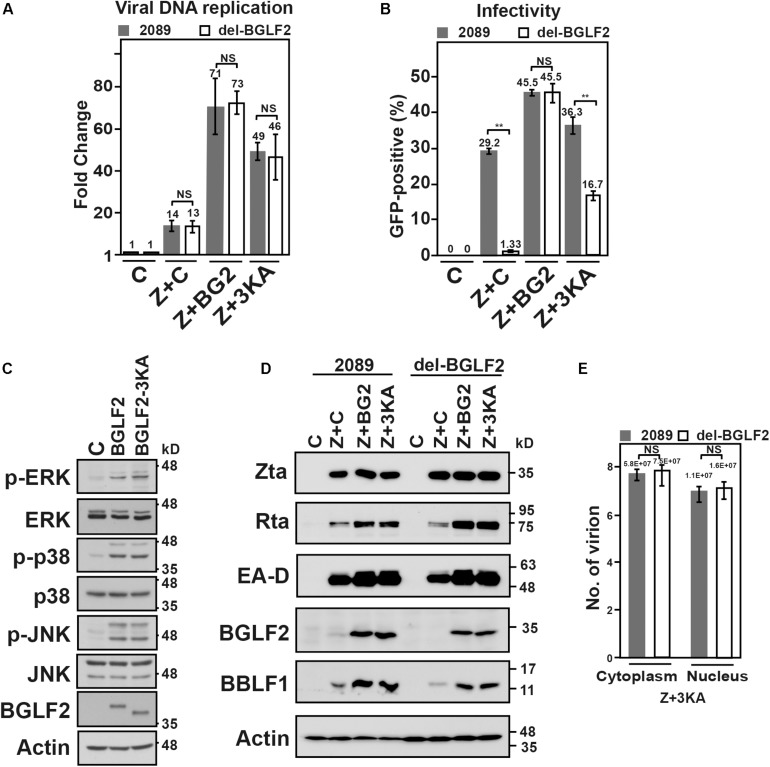
Genetic complementation of the del-BGLF2 mutation. **(A)** HEK293 (p2089) and HEK293 (del-BGLF2) cells were cotransfected with pCMV-Zta and empty vector (Z + C) to induce the Epstein–Barr virus (EBV) lytic cycle and also cotransfected with pCMV-Zta and BGLF2 (Z + BG2) or BGLF2-3KA (Z + 3KA). The amount of EBV DNA relative to that in the control cell lysate at 48 h after lytic induction was determined by qPCR using primers specific to EBNA1 gene, which was normalized against the number of copies of *PIK3CA*. **(B)** The culture media were used to infect Raji cells. On day 3 post-infection, the percentage of Raji cells that were GFP-positive was determined by FACS analysis. The experiments were conducted three times, and the results were analyzed statistically using the *t*-test. *p < 0.05; **p < 0.01; NS, not statistically significant. **(C)** 293T cells transfected with pCMV3 or plasmids that expressed V5-BGLF2 or V5-BGLF2-3KA were analyzed by immunoblotting with indicated antibodies 48 h post-transfection. **(D)** HEK293 (p2089) and HEK293 (del-BGLF2) cells were cotransfected with pCMV-Zta and empty vector (Z + C) to induce the EBV lytic cycle and also cotransfected with pCMV-Zta and BGLF2 (Z + BG2) or BGLF2-3KA (Z + 3KA). On day 3 post-transfection, proteins in the lysate were analyzed by immunoblotting with anti-Rta, anti-EA-D, anti-BGLF2, anti-BBLF1, and anti-actin antibodies. **(E)** HEK293 (2089) and HEK293 (del-BGLF2) that were cotransfected with pCMVZta and pCMV-BGLF2-3KA were lysed with 0.5% NP40 at 48 h post-transfection, and the lysate was then separated into cytoplasmic and nuclear fractions. The samples were treated with DNase I and proteinase K. The encapsidated EBV DNA was purified and quantified by qPCR. NS, not statistically significant.

To further determine if the interaction between BGLF2 and BBLF1 is important for the production of the infectious virion, BGLF2, or BGLF2-3KA, a mutant defective in the interaction with BBLF1, was exogenously expressed in cells at the time of lytic induction. As a control, in HEK293 (p2098) and HEK293 (del-BGLF2) cells that were cotransfected with pCMV-Zta (Z) and empty vector (C), EBV DNA replication increased by 14- and 13-fold (Figure 7A; Z + C), respectively, over the background on day 2. The culture medium from HEK293 (p2098) that had been transfected with pCMV-Zta and empty vector infected 29.2% of the Raji population, whereas the culture medium from HEK293 (del-BGLF2) infected only 1.33% of Raji population (Figure 7B; Z + C), which are consistent with that in Figure 6, showing that BGLF2 is required for the production of infectious viral progeny. We also cotransfected the cells with pCMV-Zta and pCMV-BGLF2, and EBV DNA replication by both cell types further increased to the same level, about 70-fold over the background (Figure 7A). As BGLF2 is an EBV late gene (Figure 1), which is not expressed at the stage of EBV lytic DNA replication. The activation observed is likely attributed to the activation of MAPK signaling by BGLF2 (Figure 7C) (Liu and Cohen, 2015), which is known to promote the expression of Zta (Liu and Cohen, 2015). Under the condition of similar levels of DNA replication after cotransfecting pCMV-Zta and pCMV-BGLF2, 45% of the green Raji population by HEK293 (p2089) and HEK293 (del-BGLF2) was detected. The result showed that defective production of infectious progeny in del BGLF2 EBV was rescued by exogenous BGLF2 (Figure 7B; Z + BG2). This study further examined whether the 3KA mutations can rescue the defect of the production of infectious virion. Before this was done, we first analyzed whether the mutations influence MAPK signaling and found that expression of BGLF2 or BGLF2-3KA increased the levels of phospho-ERK, phospho-p38, and phospho-JNK (Figure 7C), indicating that both proteins activate MAPK signaling; both enhanced Zta-activated expression of Rta, EA-D, and BBLF1 (Figure 7D). This study also found that transfecting the plasmid expressing BGLF2-3KA increased EBV DNA replication about 50-fold (Figure 7A; Z + 3KA) in both 2089 and del-BGLF2. Although the level of DNA replication is similar between 2089 and del-BGLF2 cotransfecting with pCMV-Zta and pCMV-BGLF2-3KA, viral infectivity was 16.7% in HEK293 (del-BGLF2) compared to 36.3% in HEK293 (p2089) (Figure 7B; Z + 3KA), suggesting that BGLF2-3KA partially rescues the production of infectious progeny by del-BGLF2. Furthermore, the effect of BGLF2-3KA on the viral assembly in 2089 and del-BGLF2 was investigated. Accordingly, the amounts of encapsidated EBV DNA in the nuclear and cytoplasm were determined by qPCR. We found that encapsidated EBV DNA in the nucleus or cytoplasm in HEK293 (p2089) and HEK293 (delBGLF2) cells were about equal (Figure 7E). Under the same levels of viral lytic replication, similar numbers of viral particles were found in the nucleus and cytoplasm. The results showed that BGLF2-3KA, which was expressed from a plasmid after transfection, partially rescued the production of infectious virions by del-BGLF2. The presence of about equal amounts of encapsidated DNA in both cell types after transfecting the pCMV-BGLF2-3KA (Figure 7E) suggested that the partial rescue is not due to the defect in capsid assembly and nuclear egress. Since the 3KA mutation weakens the interaction with BBLF1 (Figure 4B), it suggested that the interaction between BGLF2 and BBLF1 is important to the efficient production of infectious viral particles.

## Discussion

Viral maturation of herpesviruses is a complex process, which is regulated in a spatiotemporal manner (Diefenbach, 2015; Owen et al., 2015). This study shows that BGLF2, a tegument protein (Johannsen et al., 2004), associates with the EBV capsid before final envelopment, is recruited to TGN *via* the interaction with BBLF, and is important for the efficient production of infectious virion.

This study reveals that the expression of BGLF2 is inhibited by PAA, showing that BGLF2 is expressed in the late stage of the EBV lytic cycle after lytic DNA replication (Figure 1A). The expression of BGLF2 appears to promote EBV viral production but does not affect the other EBV lytic functions, as the knockout of the expression of BGLF2 substantially reduces the number of EBV particles that are produced by the cells but does not affect the expression of EBV genes or lytic DNA replication (Figures 6, 7). This conclusion is supported by the results of HSV-1 UL16 (Baines and Roizman, 1991; Starkey et al., 2014), HCMV UL94 (Phillips and Bresnahan, 2012), MHV-68, and KSHV ORF33 (Guo et al., 2009; Wu et al., 2015) studies, as mutating these genes attenuated or abolished viral production.

In this study, we find that BBLF1 and BGLF2 interact both *in vitro* and *in vivo* (Figures 2A,B). The NDYEE_31_ acidic cluster motif in BBLF1 is important for the interaction with BGLF2 (Figure 3). A similar acidic cluster motif in HSV-1 UL11 was also observed to be important to its interaction with UL16 (Loomis et al., 2003; Yeh et al., 2008). Our earlier study showed that PACS1 interacts with the acidic cluster domains in BBLF1; this interaction is required for the retrograde transport of BBLF1 to TGN (Chiu et al., 2012). On the way to TGN, PACS1 disassociates from BBLF1, which may allow the binding of BGLF2 to BBLF1. Additionally, this study shows that the N-terminal 155-amino acid region in BGLF2 interacts with BBLF1 (Figure 4A). This region contains a basic amino acid motif, KKK_69_; similar basic amino acid motifs are also present in the N-terminal region in UL16 homologs. Mutating KKK_69_ motif in BGLF2, BGLF2-3KA, substantially weakens its interaction with BBLF1 (Figure 4B). Immunofluorescence staining reveals that BBLF1, but not its NDE mutant, redistributed cytosolic BGLF2 to TGN (Figure 3C). In parallel, BGLF2-3KA is not redistributed from the cytosol to TGN by BBLF1 (Figure 4C; fourth row), further supporting that the KKK_69_ motif of BGLF2 and NDYEE_31_ acidic cluster motif in BBLF1 are crucial for the interaction. These results suggest that the interaction between the BBLF1 and BGLF2 is a prerequisite for targeting BGLF2 to the TGN membrane.

An earlier study showed that UL16 is partially recruited by UL11 to TGN (Loomis et al., 2003). The binding of UL21 to the C-terminal region in UL16 has been suggested to promote the recruitment of UL16 to membrane by interaction with UL11, and the lack of binding of UL21 to UL16 causes the partial recruitment (Harper et al., 2010; Chadha et al., 2012; Han et al., 2012; Diefenbach, 2015). The UL21 is known as a capsid-binding tegument. UL16 is likely to interact with UL11 until UL16 interacts with capsid-associated UL21, which leads to escort capsid to UL11-localized TGN for final envelopment. This study shows that BGLF2 is also partially recruited to TGN by BBLF1 (Figures 2–4), suggesting the interaction between BGLF2 and BBLF1 is likely regulated. However, EBV does not encode a UL21 homolog; the mechanism that regulates BGLF2 to interact with BBLF1 and is recruited to TGN is still unclear.

The 3KA mutation in BGLF2 is found to significantly weaken the interaction between BGLF2 and BBLF1 (Figure 4B), allowing us to investigate how the interaction between BGLF2 and BBLF1 influences EBV maturation. However, only a partial rescue is observed after the cells were transfected with a plasmid that encoded BGLF2-3KA (Figure 7B; Z + 3KA). An earlier study showed that BGLF2-mediated MAPK signaling promotes the production of infectious EBV (Konishi et al., 2018), and mutating the KKK motif in BGLF2 does not influence its ability to activate MAPK signaling (Figure 7C). The partial rescue may be attributed to the activation of MAPK pathway by BGLF2-3KA but a failure to interact with BBLF1. In addition, the amounts of viral particles in the nucleus and cytoplasm are about equal when BGLF2-3KA is exogenously expressed in HEK293 (p2089) and HEK293 (del-BGLF2) cells, suggesting that the reduction in the production of infectious progeny is unlikely due to the defect in capsid assembly and nuclear egress. The results suggest that the BGLF2-BBLF1 interaction is important to the efficient production of infectious EBV.

Our results showed that the deletion of BGLF2 from the EBV genome does not affect viral DNA replication (Figures 6E, 7A; Z + C), but exogenously expressed BGLF2 and BGLF2-3KA promote viral DNA replication (Figure 7A; Z + BG2 and Z + 3KA). A previous study showed that BGLF2 activates the MAPK pathway; exogenously expressed BGLF2 not only promotes the expression of endogenous BZLF1 (Liu and Cohen, 2015) but also activates promoters that contain the AP-1 site, such as CMV promoter that drives expression of Zta from a plasmid. Therefore, exogenous expression of BGLF2 that promotes EBV lytic replication is expected. However, BGLF2 activates BZLF1 expression, and lytic replication may only occur during primary infection rather than lytic reactivation, that is, by cotransfecting pCMV-Zta and pCMV-BGLF2 (Figure 7). This study finds that mutating the KKK motif in BGLF2 does not influence its ability to activate MAPK signaling (Figure 7C), but the increase in replication by BGLF2-3KA is reduced by about 30% (Figure 7A; Z + 3KA). Recent studies showed that BGLF2 influences NF-κB and JAK-STAT signaling pathway (Chen et al., 2019; Sonia et al., 2019), whether the mutation in KKK motif in BGLF2 influences these signaling pathways to affect viral DNA replication is unclear. However, as the 3KA mutations in BGLF2 does not seem to affect EBV DNA replication in HEK293 (p2089) and HEK293 (del-BGLF2) cells, this allows us to evaluate the reduction effect of BGLF2-3KA on the production of infectious progeny (Figure 7B; Z + 3KA) on the basis of similar levels of viral DNA replication.

Our study observed that BGLF2-3KA reduces the nuclear distribution in A7 cells (Figure 4C), suggesting that KKK_69_ may serve as a nuclear localization signal (NLS) of BGLF2. However, the lack of the possible NLS probably does not influence the nuclear entry of BGLF2-3KA as BGLF2-3KA is a small protein of 35 kDa; the protein is able to enter the nucleus without an NLS (Figure 4C). In addition, BGLF2 may enter the nucleus through interaction with other viral proteins, such as BKRF4 (Masud et al., 2017). Like BGLF2, BGLF2-3KA interacts with BKRF4 (data not shown). Our results showed that the amounts of viral particles in the nucleus and cytoplasm are similar between HEK293 (p2089) and HEK293 (del-BGLF2) cells when BGLF2-3KA is expressed from a plasmid, suggesting that the reduction in the production of infectious progeny is likely due to the impairment in the late stage of viral maturation in the cytoplasm. Therefore, it is unlikely that the nuclear distribution of BGLF2-3KA contributes to the reduction in the production of infectious progeny.

Density-gradient centrifugation herein showed that BGLF2 was associated with the capsid before it gained an envelope (Figure 5B). An earlier yeast two-hybrid screen found that BGLF2 is a binding partner of the EBV major capsid protein VCA (Fossum et al., 2009). It is likely that the BBLF1 interacts with BGLF2 to recruit capsids to TGN, where glycoproteins are located, for final envelopment. Without BGLF2, targeting to the TGN membrane of capsids is probably inefficient, resulting in a reduction of viral production (Figure 6G). This study reveals the function of BGLF2 in EBV maturation *via* its interaction with BBLF1, for the efficient production of infectious viral particles.

## Data Availability Statement

The datasets generated for this study are available on request to the corresponding author.

## Author Contributions

C-HH was involved in experimental design and data analysis. Y-FC, W-HW, L-WC, W-JT, and C-CY conducted the experiments. Y-FC, L-WC, P-JC, T-YH, and Y-JL discussed the experiments and data and advised the study. C-HH and Y-FC prepared the manuscript.

## Conflict of Interest

The authors declare that the research was conducted in the absence of any commercial or financial relationships that could be construed as a potential conflict of interest.
